# A human-associated *Spiroplasma ixodetis* lineage responsible for infantile cataracts and adult febrile illness

**DOI:** 10.1016/j.isci.2026.115233

**Published:** 2026-03-06

**Authors:** Marie Buysse, Matthew J. Ballinger, Marjorie Bruley, Julien Amoros, Justine Grillet, Navid Farassat, Annerose Serr, Wolf Alexander Lagrèze, Christine Wennerås, Anna Grankvist, Thomas Schön, Jonas Berglund, Lesley Bell-Sakyi, Hein Sprong, Olivier Duron

**Affiliations:** 1 MIVEGEC, University of Montpellier, Centre National de la Recherche Scientifique (CNRS), Institut de recherche pour le développement (IRD), Montpellier, France; 2 Department of Biological Sciences, Mississippi State University, Mississippi State, MS, USA; 3 Eye Center, Medical Center, University Freiburg, Freiburg, Germany; 4 Institute for Microbiology and Hygiene, Faculty of Medicine, University Freiburg, Freiburg, Germany; 5 Department of Infectious Diseases, The Sahlgrenska Academy, University of Gothenburg, Göteborg, Sweden; 6 Department of Infectious Diseases, Kalmar County Hospital, Linköping University, Kalmar, Sweden; 7 Department of Biomedical and Clinical Sciences, Linköping University, Linköping, Sweden; 8 Department of Infectious Diseases in Östergötland, Linköping University, Linköping, Sweden; 9 Department of Infection Biology and Microbiomes, Institute of Infection, Veterinary and Ecological Sciences, University of Liverpool, Liverpool, UK; 10 Centre for Infectious diseases, National Institute for Public Health and the Environment, Bilthoven, Utrecht, the Netherlands

**Keywords:** Health sciences, Medicine, Medical science, Medical specialty, Organism

## Abstract

Bacteria of the *Spiroplasma ixodetis* clade are well characterized as reproductive parasites and defensive endosymbionts of arthropods. Nevertheless, clinical evidence indicates that they can also infect humans, causing neonatal ocular disease and acute febrile illness in adults. Using metagenomic assembly and phylogenomic analyses of *Spiroplasma ixodetis*-related human infections (SiRHIs), combined with a systematic meta-analysis of public datasets, we identified 25 human cases across ten European countries. Despite the frequent detection of multiple *S*. *ixodetis* strains in ticks, our data provide no evidence implicating tick-associated strains in human infections. Instead, SiRHI constitute a distinct monophyletic lineage within the *S*. *ixodetis* clade, consistent with a shared evolutionary origin with arthropod-associated relatives. Notably, SiRHI genomes harbor horizontally acquired chaperone genes absent from most arthropod-associated *Spiroplasma*, while retaining conserved effector genes typical of endosymbionts, suggesting the preservation of ancestral symbiotic traits alongside newly acquired molecular adaptations.

## Highlights

• Genomic and database analyses identify 25 human infections caused by SiRHI bacteria
• SiRHI represents a distinct lineage within the *Spiroplasma ixodetis* clade
• Horizontally acquired chaperones and conserved effectors support SiRHI’s infection potential

## Graphical abstract

**Figure:**
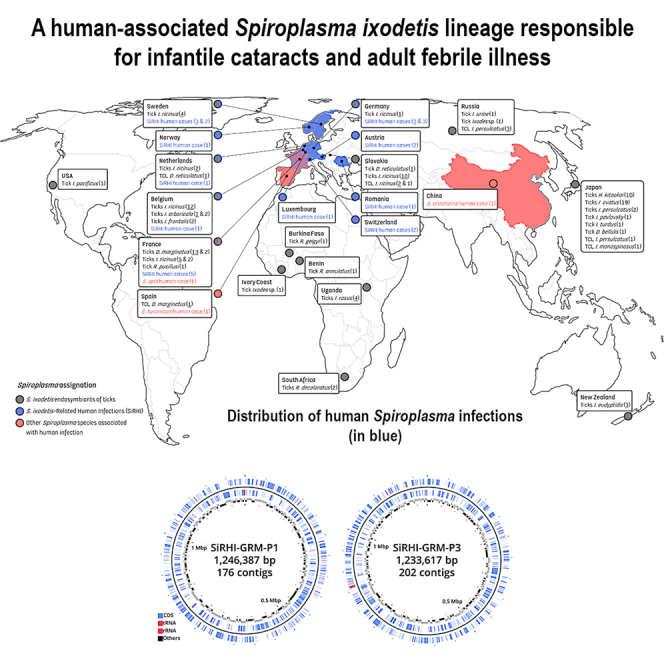

## Introduction

Ocular disorders in neonates and infants, associated with *Spiroplasma* bacterial infections, have been sporadically documented for at least three decades across Europe.^1^^,^^2^^,^^3^^,^^4^^,^^5^^,^^6^ Initially identified in 1996 with a congenital inflammatory cataract case in a 4-month-old premature baby in Germany,^1^ similar infections remained undocumented for two decades. However, a retrospective survey in France in 2020 described three new cases of congenital cataracts and ocular inflammations associated with *Spiroplasma*.^2^ This report was followed by two additional cases identified in Germany in 2021,^3^ and one recent case in Luxembourg in 2023,^6^ along with another in Belgium in 2024.^5^ In 2025, a new survey documented 11 additional unpublished cases presenting similar symptoms, occurring between 2009 and 2022 across Germany, Austria, Norway, France, Romania, the Netherlands, and Switzerland.^4^ Remarkably, systemic infections by *Spiroplasma* have also surfaced in adults since 2015, presenting as acute febrile illness in Switzerland,^7^ Spain,^8^ France,^9^ Sweden,^10^ and, more recently, in China.^11^ Members of the *Spiroplasma* genus were confirmed as the etiological agents in these cases, as no alternative microbial pathogens or causes could be identified. However, the global burden of *Spiroplasma*-induced diseases on human health remains unclear, as current knowledge is primarily based on sporadic and opportunistic detection of infection cases, which required curative antibiotic treatment, and in some cases, surgical intervention and hospitalization.^1^^,^^2^^,^^3^^,^^5^^,^^6^^,^^7^^,^^8^^,^^9^^,^^10^^,^^11^ The origin of *Spiroplasma* infections in humans remains unknown, though tick exposure during summer months has been proposed as a potential route of transmission.^4^^,^^10^

*Spiroplasma* is a genus of cell wall-deficient, helical Mollicutes bacteria that are typically not associated with human disease or the human microbiome.^12^^,^^13^^,^^14^^,^^15^ The genus is divided into over 40 recognized species, many of which are specialized symbionts or pathogens of arthropods, while others infect plants and cause crop diseases.^12^^,^^13^^,^^14^^,^^15^ To date, only one species, *Spiroplasma mirum*, originally isolated from ticks, has been experimentally demonstrated to infect vertebrate hosts, though this has been observed exclusively in neonate or suckling laboratory rodents, whereas adults of the same species were refractory to infection.^16^^,^^17^^,^^18^^,^^19^ A few studies have also speculated on the possible involvement of *S*. *mirum* in transmissible spongiform encephalopathies,^20^^,^^21^ but definitive evidence remains lacking.^22^ Amid this diversity, the taxonomic identification of *Spiroplasma* species and strains involved in human infections remains uncertain, although most cases have been confirmed through gene sequencing.^1^^,^^2^^,^^3^^,^^5^^,^^6^^,^^7^^,^^8^^,^^9^^,^^10^^,^^11^ Indeed, all studies have relied exclusively on 16S rRNA gene sequence as a single genetic marker for molecular identification, with some infections tentatively attributed to *Spiroplasma ixodetis*.^1^^,^^2^^,^^3^^,^^7^^,^^10^ However, this assignment remains challenging, as 16S rRNA sequences are highly conserved and often nearly identical among closely related bacterial species. Additionally, comparative analyses of *Spiroplasma* infections in humans are further hampered by methodological inconsistencies across studies, including variation in typing protocols and the use of short, non-overlapping fragments from different regions of the 16S rRNA sequence.^1^^,^^2^^,^^3^^,^^7^^,^^8^^,^^9^^,^^10^^,^^11^

Members of the *S*. *ixodetis* clade are predominantly recognized as highly specialized endosymbionts exclusively associated with arthropod hosts, characterized by transovarial transmission and complex effects favoring their spread in arthropod populations.^13^^,^^15^ Certain strains are beneficial defensive endosymbionts, protecting their insect hosts against entomopathogenic fungi.^23^ Other strains are reproductive parasites of insects, inducing either male-specific mortality during embryogenesis (male-killing, MK),^24^^,^^25^^,^^26^^,^^27^^,^^28^^,^^29^ or sperm-mediated mortality in uninfected embryos (cytoplasmic incompatibility, CI).^30^ The *S*. *ixodetis* type strain (Y32 strain) was initially characterized in 1981 in western black-legged ticks, *Ixodes pacificus*, from Oregon, US.^31^^,^^32^ Subsequent studies have detected members of the *S*. *ixodetis* clade in over ten tick species across Europe, Africa, and Asia.^33^^,^^34^^,^^35^^,^^36^^,^^37^^,^^38^^,^^39^^,^^40^^,^^41^ However, despite its species name (referring to its original association with an ixodid tick), *S*. *ixodetis* is not exclusive to ticks and commonly infects a broad range of arthropods, including other arachnids such as spiders and mites, as well as insects belonging to various orders, including Hymenoptera, Lepidoptera, Diptera, Coleoptera, Hemiptera, and Odonata.^24^^,^^25^^,^^28^^,^^38^^,^^42^^,^^43^^,^^44^^,^^45^

Whether *Spiroplasma* strains infecting humans represent zoonotic transmission from arthropods, distinct evolutionary lineages, or both, remains elusive. Persistent uncertainty also concerns the accurate classification of human *Spiroplasma* infections as *S*. *ixodetis* and their possible genetic relationship to MK and CI reproductive manipulator lineages associated with arthropods. These uncertainties primarily stem from the lack of high-resolution genetic typing of *Spiroplasma* strains infecting humans, thereby limiting the ability to accurately trace evolutionary and ecological relationships of bacterial strains. Additionally, the genome of some *Spiroplasma* species,^46^ like those of some other Mollicutes,^47^ contains two genetically distinct 16S-23S-5S rRNA operons, which can complicate taxonomic assignment and downstream analyses when relying solely on the 16S rRNA genetic marker.

Accurate identification of *Spiroplasma* strains responsible for human infections remains a major challenge, underscoring the need for advanced genomic approaches to elucidate their role in human disease and potential zoonotic transmission. In this study, we address this issue through a comprehensive genetic analysis of published human cases, supplemented by newly obtained near-full-length 16S rRNA sequences from biopsies of patients, including neonates and infants with ocular disorders, and blood samples from adults with acute febrile illness. We then performed a multi-locus sequence analysis of *Spiroplasma* strains, including those from medically relevant tick species and a variety of other arthropod hosts, to place human infections within a robust phylogenetic framework and compare them to arthropod-associated *Spiroplasma* strains. Additionally, we assembled genomes from metagenomic data derived from patient samples, comparing them to existing *Spiroplasma* genomes to uncover distinctive metabolic profiles associated with human-infecting strains.

## Results

### Molecular typing of human-associated *Spiroplasma*

**Figure 1:**
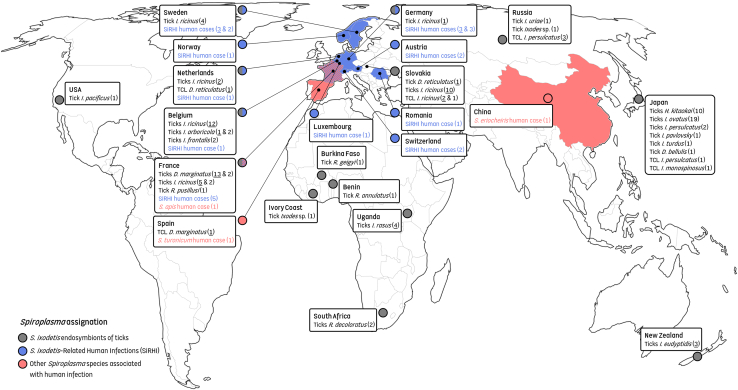
Distribution of human *Spiroplasma* infections and origin of tick samples analyzed in this study The color and label of each circle represent the sample type: blue for SiRHIs, red for those linked to other *Spiroplasma* species, gray for tick samples. Samples newly produced or newly analyzed for this study are underlined. TCL refers to a tick cell line.

We conducted an initial investigation of samples from six European patients with diagnosed *Spiroplasma* infections, including two infants and one neonate from Germany (referred to as GRM-P1, GRM-P2, and GRM-P3) diagnosed early with inflammatory cataract,^3^^,^^4^ as well as three adults from Sweden (SWD-P1, SWD-P2, and SWD-P3) with acute febrile illness, reported here as novel cases (Tables 1 and S1; Figure 1). Initial identification of these *Spiroplasma* infections relied solely on a short 16S rRNA gene fragment (Tables 1 and S1). We then performed multi-locus typing using *Spiroplasma*-specific primers^38^ to amplify extended fragments of 16S rRNA (1,248 bp), along with fragments of the genes encoding the RNA polymerase β-subunit (*rpoB*; 543–546 bp), DNA gyrase subunit A (*gyrA*; 517–518 bp), chaperone protein DnaK (*dnaK*; 418 bp), and transmembrane protein EPSG (*EpsG*; 466 bp). Importantly, these genes are conserved housekeeping and surface protein-encoding loci that offer greater phylogenetic resolution than 16S rRNA alone while remaining stable enough to allow robust comparisons across *Spiroplasma* strains.^38^ Gene fragment sequences for *Spiroplasma rpoB*, *gyrA*, *dnaK*, and *EpsG* were obtained from all six patients, while 16S rRNA sequences were successfully recovered from all the patients apart from one German infant due to poor DNA quality (Table S3). This multi-locus typing confirmed that the six patients were infected with bacteria belonging to the *S*. *ixodetis* clade, which we designate as *Spiroplasma ixodetis*-related to human infections (SiRHIs), as detailed below. Additionally, amplification of the 16S rRNA fragment yielded clean, unambiguous sequences without double peaks, consistent with the presence of a single rRNA operon for each *Spiroplasma* infection case, as further confirmed by metagenomic analysis.

**Table 1:** List and major features of reported SiRHIs

*Spiroplasma* isolate	Primary identification	Case geographic origin	Year of occurrence	Patient	Major symptoms	Position of the sequenced 16S rRNA fragment∗	Reference
SiRHI-GRM-P1	*Spiroplasma* sp.	Germany	2016	3-month-old boy	Ocular	72–1,477 (1,406 bp)	Farassat et al.^3^ 2021
SiRHI-GRM-P2	*S*. *ixodetis*	Germany	2020	3-week-old premature child	Ocular	ND	Farassat et al.^3^ 2021
SiRHI-GRM-P3	*S*. *ixodetis*	Germany	2021	3-day-old child	Ocular	53–1,471 (1,419 bp)	Os et al.^4^ 2025
SiRHI-SWD-P1	*Spiroplasma* sp.	Kalmar, Sweden	2024	77-year-old male	Systemic	107–1,469 (1,363 bp)	This study
SiRHI-SWD-P2	*Spiroplasma* sp.	Kalmar, Sweden	2024	52-year-old male	Systemic	107–1,469 (1,363 bp)	This study
SiRHI-SWD-P3	*Spiroplasma* sp.	Kalmar, Sweden	2024	75-year-old male	Systemic	107–1,469 (1,363 bp)	This study
Unnamed	*S*. *ixodetis*	Germany	1996	4-month-old premature child	Ocular	1,190–1,390 (201 bp)	Lorenz et al.^1^ 2022
Unnamed	*Spiroplasma* sp.	Giessen, Germany	2009	6-month-old child	Ocular	ND	Os et al.^4^ 2025
Unnamed	*Spiroplasma* sp.	Giessen, Germany	2010	8-month-old premature child	Ocular	ND	Os et al.^4^ 2025
Unnamed	*Spiroplasma* sp.	Vienna, Austria	2012	3-month-old girl	Ocular	ND	Os et al.^4^ 2025
Unnamed	*Spiroplasma* sp.	Austria	2013	3-month-old girl	Ocular	ND	Os et al.^4^ 2025
14_00057	*S*. *ixodetis*	France	2014	26-day-old girl	Ocular	95–1,400 (1,306 bp) (MN207005)	Matet et al.^2^ 2020
18_00012	*S*. *ixodetis*	France	2018	3-day-old boy	Ocular	100–1,383 (1,284 bp) (MN166760)	Matet et al.^2^ 2020
19_00020	*S*. *ixodetis*	France	2019	1-month-old boy	Ocular	100–1,350 (1,251 bp) (MN166762)	Matet et al.^2^ 2020
OUS	*S*. *ixodetis*	Norway	2019	3-day-old girl	Ocular	56–693 (638 bp) (PQ451577)	Os et al.^4^ 2025
21_00006	*S*. *ixodetis*	France	2020	10-day-old boy	Ocular	ND	Os et al.^4^ 2025
Clone 558-2533-21	*S*. *ixodetis*	Kalmar, Sweden	2021	81-year-old woman	Systemic	47–748 (702 bp) (OL636349)	Eimer^10^ 2022
Clone 558-4107-21	*S*. *ixodetis*	Visby, Sweden	2021	76-year-old man	Systemic	40–747 (708 bp) (OL636350)	Eimer^10^ 2022
Unnamed	*S*. *ixodetis*	Bucharest, Romania	2022	2-week-old girl	Ocular	ND	Os et al.^4^ 2025
22_00014	*S*. *ixodetis*	France	2022	16-day-old girl	Ocular	ND	Os et al.^4^ 2025
Unnamed	*S*. *ixodetis*	Luxembourg	ND	19-day-old boy	Ocular	ND	Martin et al.^6^ 2023
Unnamed	*S*. *ixodetis*	Belgium	ND	1-month-old girl	Ocular	ND	Van Haecke et al.^5^ 2024
Clone Zurich	*S*. *ixodetis*	Switzerland	ND	70-year-old woman	Systemic	10–526 (517 bp) (KJ639831)	Mueller et al.^7^ 2015
Unnamed	*Spiroplasma* sp.	Netherlands	ND	11-week-old girl	Ocular	ND	Os et al.^4^ 2025
Unnamed	*S*. *ixodetis*	Switzerland	ND	Premature baby girl	Ocular	ND	Os et al.^4^ 2025

Isolates SiRHI-GRM-P1 to GRM-P3 and SiRHI-SWD-P1 to SWD-**P3** are from the six patients analyzed in this study. ND, not determined. ∗, Position and accession numbers of the sequenced 16S rRNA fragment relative to the full 16S rRNA sequence of *S*. *ixodetis* strain Y32 reference sequence.

A single strain, designated SiRHI-SWD, was identified in all three Swedish adult patients, based on identical *Spiroplasma* sequences for the 16S rRNA, *rpoB*, *gyrA*, *dnaK*, and *EpsG* genes. In contrast, while the neonate and two infants from Germany shared identical *rpoB*, *gyrA*, *dnaK*, and *EpsG* sequences, a slight variation was observed in the 16S rRNA gene between the GRM-P1 and GRM-P3 cases (99.85% identity; 1,360/1,362 bp; Table S2). Consequently, we distinguish between these two strains, designated SiRHI-GRM-P1 and SiRHI-GRM-P3. Although no sequence variation was observed in the *rpoB*, *gyrA*, *dnaK*, and *EpsG* genes, SiRHI-SWD, SiRHI-GRM-P1, and SiRHI-GRM-P3 differed in their 16S rRNA sequences (99.56% identity between SiRHI-SWD and SiRHI-GRM-P1; 99.71% between SiRHI-SWD and SiRHI-GRM-P3, over 1,362 bp). All three SiRHI strains exhibited high 16S rRNA sequence identity with the *S*. *ixodetis* Y32 type strain, which carries two rRNA operons, yet they remain clearly distinct from it (*S*. *ixodetis* Y32 operon #1: 98.14%, 98.28%, and 98.60% over 1,394 bp for SiRHI-GRM-P1, SiRHI-GRM-P3 and SiRHI-SWD; *S*. *ixodetis* Y32 operon #2: 98.85%, 99.00%, and 99.27% over 1,362 bp; Table S2). The 16S rRNA sequences of the three SiRHI strains displayed substantially lower identities with other species of the *S*. *ixodetis* clade (*S*. *platyhelix* [GCA_021496725]: 93.49%, 93.65% and 93.73% over 1,242 bp) and more distantly related species (*S*. *mirum*: 87.69%, 87.86%, and 87.94%; *S*. *citri*: 86.85%, 87.02%, and 87.10%; *S*. *poulsonii* [GCA_000820525]: 86.43%, 86.60%, and 86.68%, over 1,242 bp; Table S2).

**Figure 2:**
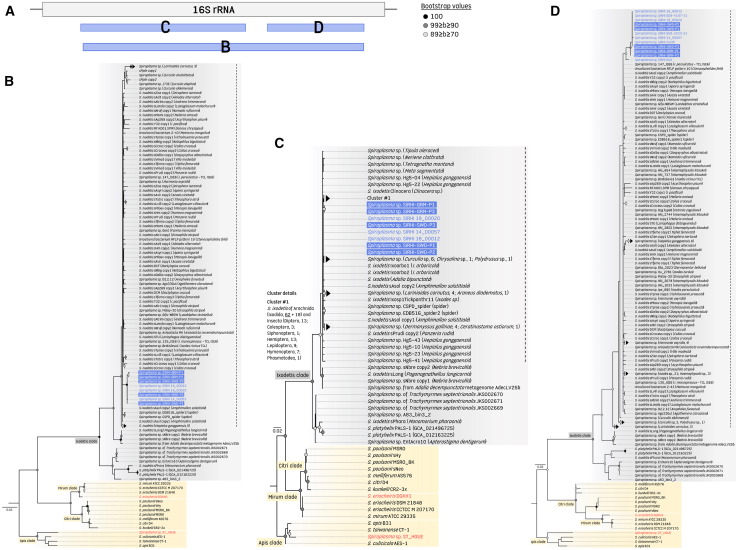
Phylogenetic assignment of human *Spiroplasma* infections based on 16S rRNA gene sequences Previous studies of *Spiroplasma* infections in humans have used different 16S rRNA typing protocols, and most of the sequences available in GenBank were obtained from short and distinct positions along the 16S rRNA gene sequence (listed in Table S1). As a result, most of these short 16S rRNA gene sequences cannot be used in the same phylogenetic analysis because they do not overlap and cannot be aligned with each other. To circumvent this issue and include all *Spiroplasma* sequences from humans in phylogenetic analyses, we thus created three separate datasets each based on (A) the three regions along the 16S rRNA gene sequence, including (B) 1,165 unambiguously aligned bp with a GTR + G4 + I model, (C) 715 unambiguously aligned bp with an HKY + G4 model, and (D) 383 unambiguously aligned bp with an HKY + G4 model. Phylogenetic trees were constructed using maximum-likelihood estimation with 1,000 bootstrap replicates. Only bootstrap values greater than 70% are shown. The scale bars represent substitutions per site. Sequences generated in this study are underlined. Specifically, *Spiroplasma* sequences from SiRHIs are shown in blue text, SIRHI sequences produced in this study are highlighted in blue, while other *Spiroplasma* species associated with reported human cases are shown in red text. The dashed line indicates the proposed limit delineating the *S*. *ixodetis* species. GenBank accession numbers and sequence origins are provided in Tables S3, S4, and S5. TCL refers to a tick cell line.

The five near-full-length 16S rRNA gene sequences obtained in the present study were used to compare the SiRHI strains with other published *Spiroplasma* infections diagnosed on the sole basis of sequences from distinct 16S rRNA regions (Tables 1 and S1). This comparison revealed no or extremely low nucleotide variation between the SiRHI-GRM-P1, SiRHI-GRM-P3, and SiRHI-SWD strains and eight other human cases (Table S2), including (i) in Germany, a premature baby with cataracts (SiRHI-GRM-P1 & SiRHI-GRM-P3: 98.76%, 159/161 bp; SiRHI-SWD: 100% identity, 161 bp),^1^ (ii) in France, three neonates and infants with cataracts and ocular inflammations (SiRHI-GRM-P1 & SiRHI-GRM-P3: 99.68%–99.76%, 1,240-1,241/1,244 bp; SiRHI-SWD: 99.92%–100% identity, 1,243-1,244/1,244 bp),^2^ (iii) in Norway, a 3-day-old neonate with cataracts (SiRHI-GRM-P1 & SiRHI-GRM-P3: 98.58%; 624/633 bp; SiRHI-SWD: 98.28% identity, 572/582 bp),^4^ (iv) in Switzerland, a 70 year-old immunocompromised patient with fever, nausea, abdominal pain, peripheral edema and pronounced weakness (SiRHI-SWD: 100% identity; SiRHI-GRM: 99.76%; 419–420/420 bp),^7^ and (v) in Sweden, an 81-year-old woman with no notable medical history and a 76-year-old man with insulin-dependent type 2 diabetes and Crohn’s disease, both of whom developed systemic disease with fever, headache, and fatigue (SiRHI-GRM-P1 & SiRHI-GRM-P3: 99.53%–99.84%, 638–640/641bp; SiRHI-SWD: 99.69%–100% identity, 639–641/641 bp).^10^ While reports of eight cases of neonatal/infant infection did not publish any genetic sequences despite using 16S rRNA PCR assays,^4^ *S*. *ixodetis* was identified as the causative agent using lens biopsies for microbial culture for two cases^4^^,^^6^ and broad-range 16S rRNA sequencing for one case in Belgium.^5^ In these 11 cases, further characterization as SiRHI is not possible, but is highly probable. Including the present study and previous case reports,^1^^,^^2^^,^^3^^,^^4^^,^^5^^,^^6^^,^^7^^,^^10^ the total number of cases of human infection attributed to SiRHI thus rises to 14 (probable 25), reported from ten European countries (Tables 1 and S1). These cases appear entirely independent, differing in their date of detection, country of origin, and without geographic proximity between patients (Tables 1 and S1). As most previous studies sequenced short and non-overlapping regions of the 16S rRNA gene (Table S1), phylogenetic analyses were based on alignments of three distinct 16S rRNA sequence regions, with each placing these infection cases within the *S*. *ixodetis* clade (Figures 2A–2D).

Other *Spiroplasma* species have been sporadically reported in to infect humans, including *S*. *turonicum* (in an adult in Spain),^8^ *S*. *eriocheiris* (in an adult in China),^11^ and *S*. *apis* (in an adult in France, although no sequence data have been deposited in public databases)^9^ (Figure 1; Table S1). These three *Spiroplasma* species are phylogenetically distinct from the *S*. *ixodetis* clade, as supported by pairwise 16S rRNA nucleotide identities below 89% when compared to both the SiRHI strains and the *S*. *ixodetis* type strain Y32, as well as by phylogenetic analyses (Figures 2A–2D).

### Symptoms and SiRHI strains variation

Of the 25 probable SiRHI-associated infections, 19 cases across nine countries involved ocular manifestations in neonates and infants, while six cases from two countries were reported in adults with more systemic and diverse clinical symptoms (Tables 1 and S1). SiRHI infections have affected individuals of diverse ages and health statuses, including premature neonates, immunocompromised patients, and otherwise healthy infants and adults. To date, tick exposure has not been consistently linked to infection onset, having been reported in two of six cases with systemic symptoms and two of 17 with ocular manifestations (Table S1). Regardless of clinical presentation, no fatalities have been reported, and all infections resolved with treatment, although some cases experienced relapse.^4^ Among neonatal cases diagnosed postnatally, despite difficulties in precisely determining the timing of infection, all pregnancies resulted in live births, even if rare cases involved preterm birth.

While 16S rRNA sequence typing of lens or vitreous aspirate samples in ocular cases and blood samples in systemic cases was the most common diagnostic tool (used in 24 out of 25 cases, Table S1), two additional methods have proven effective in cases with ocular symptoms: transmission electron microscopy (TEM), used in seven out of 19 cases (detecting the pathogen in six), and microbial cultures, performed in six cases (with detection in five). However, variation in SiRHI 16S rRNA sequences does not appear to correlate with clinical presentation or patient age. For example, the 16S rRNA sequences from two infants with cataracts in France^2^ are strictly identical to those from three adults with systemic infections in Sweden (this study; 100% identity over 1,244 bp). Similarly, the sequence from a 3-day-old neonate with cataracts in Norway^4^ exhibits comparable identity to both an ocular strain (SiRHI-GRM, 98.58%) and a systemic strain (SiRHI-SWD, 98.28%), while SiRHI-GRM and SiRHI-SWD are more closely related to each other (99.56%–99.71%). Notably, highly similar yet distinct sequences have also been observed among strains associated with similar clinical manifestations. For instance, three pediatric ocular cases in France share 99.92%–100% identity each other (1,243–1,244/1,244 bp),^2^ whereas two systemic adult cases from Sweden show 99.69% identity (639/641 bp).^10^ The limited availability of sequences relative to the total number of reported cases, coupled with the lack of overlap in the sequenced 16S rRNA regions, hinders any robust assessment of geographic structuring in sequence divergence.

### Screening of SiRHI strains in ticks and other arthropods

We compared the SiRHI-GRM and SiRHI-SWD strains to *S*. *ixodetis* strains previously identified in 100 naturally infected arthropod species. This comparison included 22 tick species for which at least one *S*. *ixodetis* gene sequence (16S rRNA, *rpoB*, *dnaK*, *gyrA*, or *EpsG*) was either generated in this study (Table S3) or obtained from public databases (Tables S4 and S5). Although the geographic origins of these specimens do not correspond to the regions where human cases have been reported, the tick collection encompassed a broad taxonomic and geographic diversity. Specimens were selected based on prior detection of *S*. *ixodetis* and included species of medical and veterinary relevance (*Ixodes ricinus*, *Ixodes persulcatus*, *Ixodes pacificus*, *Dermacentor marginatus*, *Dermacentor reticulatus*), as well as species primarily associated with wildlife (e.g., *Ixodes uriae*, *Ixodes arboricola*, *Ixodes frontalis*), sampled across Europe and other continents (Tables S3, S4, and S5). To complement these data, we also incorporated *S*. *ixodetis* sequences from 78 other arthropod species available from public databases (Tables S4 and S5). Additionally, we included sequences of related species belonging to the *S*. *ixodetis* clade, such as *S*. *platyhelix* and unnamed *Spiroplasma* species (Tables S4 and S5). On this genetic basis, none of the *S*. *ixodetis* strains identified in the 100 arthropod species were 100% identical to the SiRHI-GRM or SiRHI-SWD strains.

The SiRHI strains show the closest match to an *S*. *ixodetis* strain characterized from an undetermined *Ixodes* species collected from a leopard (*Panthera pardus*) in Ivory Coast in 1994 (Table S4), with nucleotide identities of 98.74%–98.78% to the SiRHI-GRM-P1, SiRHI-GRM-P3 and SiRHI-SWD strains, respectively, across the 16S rRNA, *rpoB*, *dnaK*, *gyrA*, and *EpsG* gene dataset (2,670 and 2,671/2,704 bp). The second and third closest matches are *S*. *ixodetis* strains identified in phytophagous flies of the family Dolichopodidae: *Thrypticus tarsalis*, collected in France (97.93%–98.04% nucleotide identity with SiRHI-GRM-P1, SiRHI-GRM-P3, and SiRHI-SWD), and *Ethiromyia chalybea*, collected in Belgium (98.00%–98.11%) (Table S4).

### Phylogeny of SiRHI strains and other members of the *Spiroplasma ixodetis* clade

**Figure 3:**
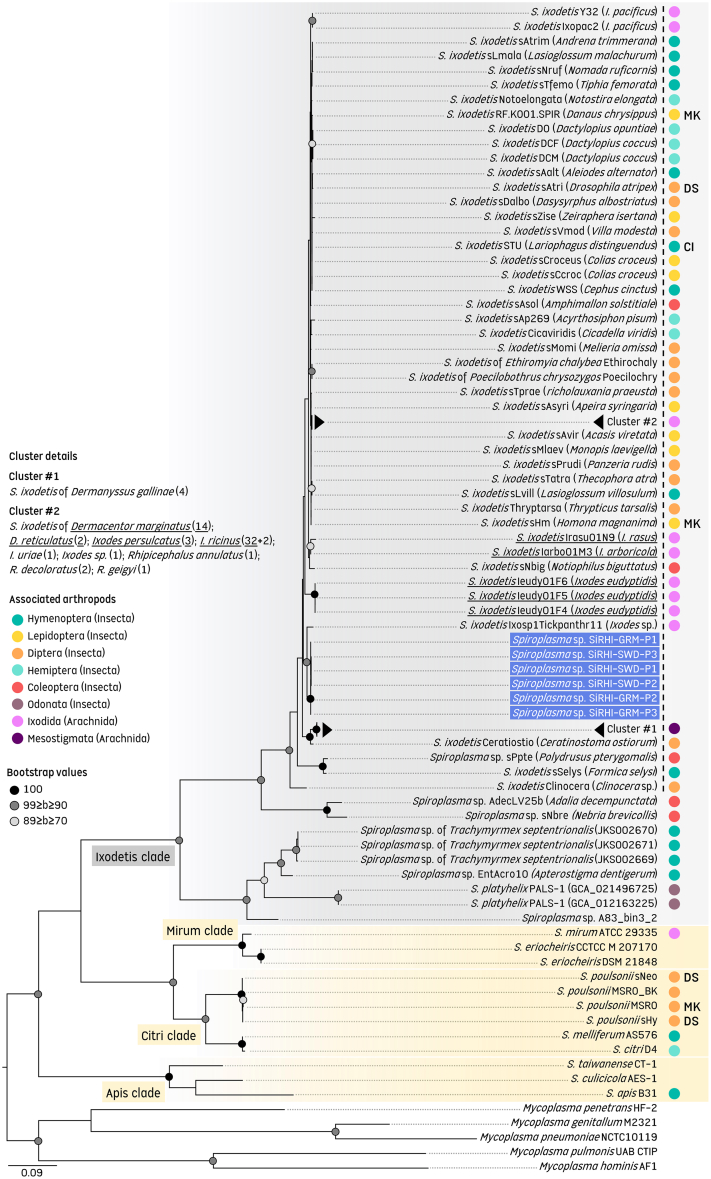
Phylogenetic relationships between SiRHI strains and other *Spiroplasma* strains and species based on multi-locus analysis The phylogeny was constructed using maximum-likelihood estimation with 1,000 bootstrap replicates, based on concatenated sequences of three genes (*dnaK*, *gyrA*, and *rpoB*; 1,478 unambiguously aligned bp, GTR + G4 + I model). Only bootstrap values greater than 70% are shown. The scale bars represent substitutions per site. Sequences generated from ticks in this study are underlined, while sequences from SiRHIs obtained in this study are highlighted in blue. Biological traits associated with *Spiroplasma* isolates, such as arthropod hosts and phenotypes (if known), are indicated on the phylogenetic tree (CI, cytoplasmic incompatibility; MK, male-killing; DS, defensive symbiosis). The dashed line indicates the proposed limit delineating the *S*. *ixodetis* species. GenBank accession numbers for each gene sequence, along with details of the origins of the sequences used, are provided in Tables S3, S4, and S5.

Phylogenetic analyses based on the multi-locus dataset revealed a common evolutionary origin of the SiRHI-GRM and SiRHI-SWD strains within the *S*. *ixodetis* clade. To maximize the number of *Spiroplasma* strains and host species included in the analysis, we examined the evolutionary relationships among *S*. *ixodetis* clade members using a partial multi-locus dataset based on the *dnaK*, *gyrA*, and *rpoB* genes (1,478 unambiguously aligned bp), since not all markers were available for every strain (Tables S3 and S4). Phylogenetic reconstructions, using maximum likelihood (ML) analyses, consistently showed that *S*. *ixodetis* clade strains form a robust monophyletic clade, including *S*. *ixodetis*, *S*. *platyhelix* and the SiRHI-GRM-P1, SiRHI-GRM-P3, and SiRHI-SWD strains, distinct from other *Spiroplasma* species (Figure 3). The SiRHI-GRM-P1, SiRHI-GRM-P3, and SiRHI-SWD strains cluster together (bootstrap value: 100) and are phylogenetically close to, but distinct from, strains of *S*. *ixodetis* found in ticks and other arthropods. Phylogenetic reconstructions also confirmed that the closest relative of the SiRHI strains is the *S*. *ixodetis* strain of the undetermined *Ixodes* species collected from an African leopard (see earlier). However, the taxonomic position of the SiRHI-GRM-P1, SiRHI-GRM-P3, and SiRHI-SWD strains remains unclear: although both clearly fall within the *S*. *ixodetis* clade, their phylogenetic position challenges clear-cut species classification and definitive assignment to the species *S*. *ixodetis* (Figure 3). Indeed, the taxonomic position of the SiRHI-GRM-P1, SiRHI-GRM-P3, and SiRHI-SWD strains suggests either that the *S*. *ixodetis* species boundary should be reconsidered to include the SiRHI strains or that the SiRHI strains represent a closely related, yet distinct, novel *Spiroplasma* species within the *S*. *ixodetis* clade.

### SiRHI metagenomics and phylogenomics

**Figure 4:**
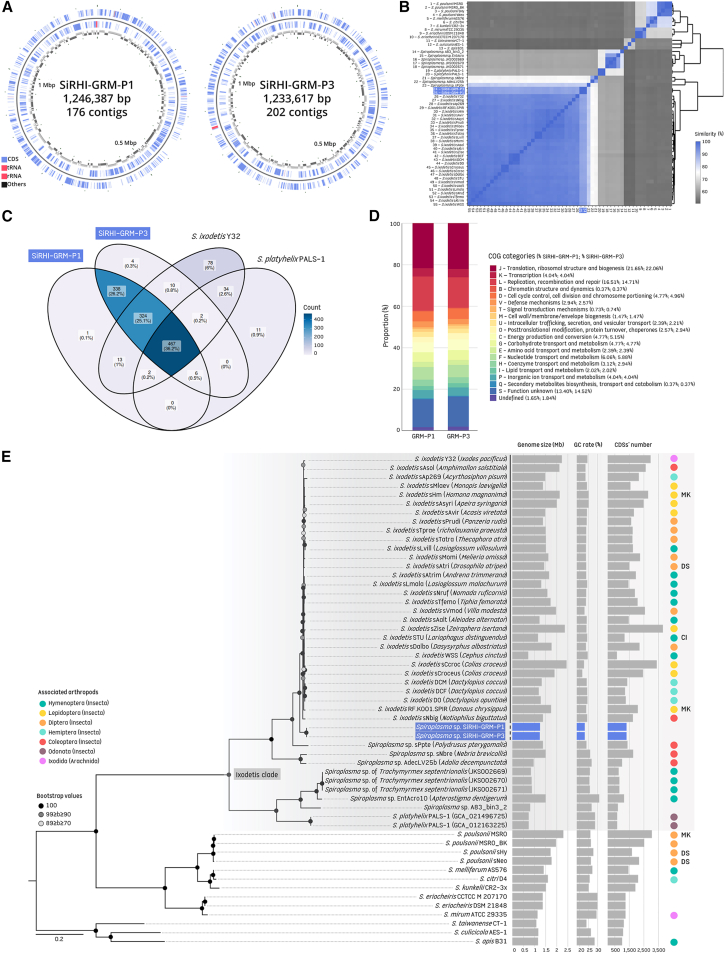
Comparative genomic features of SiRHI-GRM-P1 and SiRHI-GRM-P3 MAGs (A) Genome map of SiRHI-GRM-P1 and SiRHI-GRM-P3 MAGs. The circles on the genome maps represent the following (from outer edge to center): (1) forward strand genes, (2) reverse strand genes, and (3) contigs, depicted in gray and black. (B) Amino acid identity matrix with values represented by color, accompanied by a cladogram based on these values. SiRHI-GRM-P1 and SiRHI-GRM-P3 MAGs are highlighted in blue. The number of CDSs used to compute identity values across each genome pair ranges from 197 to 2,715. (C) Venn diagram showing the distribution of orthologs between SiRHI-GRM-P1, SiRHI-GRM-P3, *S*. *ixodetis* strain Y32 (GCA_030316605), and *S*. *platyhelix* strain PALS-1 (GCA_021496725). (D) Gene categories for SiRHI-GRM-P1 and SiRHI-GRM-P3 MAGs. Barplots were produced according to the assignment of 561 and 557 queries from the SiRHI-GRM-P1 and SiRHI-GRM-P3 MAGs, respectively. (E) Phylogenetic relationships inferred from whole-genome data using maximum likelihood (ML) based on a concatenated alignment of 86 single-copy orthologs (26,303 amino acids; best-fit evolutionary model: LG + I + G4). Numbers on each node represent bootstrap support percentages from 1,000 replicates, with only values greater than 70% shown. The scale bars correspond to the mean number of substitutions per site. SiRHI-GRM-P1 and SiRHI-GRM-P3 MAGs are highlighted in blue. Main genomic features and biological traits associated with *Spiroplasma* isolates, such as arthropod hosts and phenotypes (if known), are indicated on the phylogenetic tree (CI, cytoplasmic incompatibility; MK, male-killing; DS, defensive symbiosis). The solid line indicates the proposed delineation of the *S*. *ixodetis* species, whereas the dashed line reflects a potential extension of this delineation.

We attempted to directly sequence metagenomes from the six patient biosamples, but good quality *Spiroplasma* sequences were only obtained from lensectomies of an infant (SiRHI-GRM-P1) and a neonate (SiRHI-GRM-P3). The Metagenome-Assembled Genomes (MAGs) of SiRHI-GRM-P1 and SiRHI-GRM-P3 (with 176 and 202 contigs, respectively) are quite similar in size (1,246,387 and 1,233,617 Mb), number of predicted protein-coding genes (1,317 and 1,312) and pseudogenes (108 and 107), and having no prophage or plasmid (Figures 4A–4E; Table S6). Each SiRHI genome harbored a single 16S-23S-5S rRNA operon. We further performed additional comparative analyses using other *Spiroplasma* genomes, including all publicly available *S*. *ixodetis* genomes and unpublished genomes retrieved from the Darwin Tree of Life genomic dataset (Table S5). On this basis, both SiRHI-GRM-P1 and SiRHI-GRM-P3 exhibit moderately high estimated completeness (>77%), which may account for their smaller genome sizes and lower numbers of predicted protein-coding genes compared to those observed in *S*. *ixodetis* genomes associated with arthropods (Figure 4E; Tables S5 and S6).

**Figure 5:**
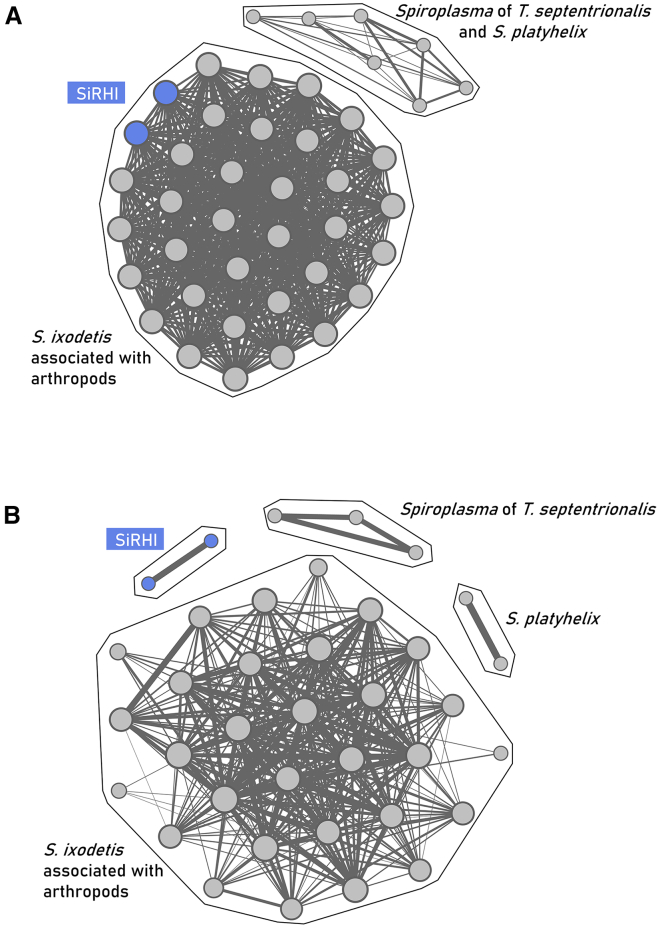
Frutcherman Reingold network analysis of *Spiroplasma ixodetis* clade genomes (A) Average amino acid identity (AAI) with edge weights >65% similarity and the (B) average nucleotide identity (ANI) with edge weights >95% similarity. The node circle diameter is proportional to the number of connections, while the edge thickness reflects the percentage of identity shared between two nodes.

Average nucleotide identity (ANI) is 99.60% between SiRHI-GRM-P1 and SiRHI-GRM-P3. Both have ANIs ranging from 76.76% to 91.92% with members of the *S*. *ixodetis* clade, including the *S*. *ixodetis* Y32 type strain (SiRHI-GRM-P1: 90.76%; SiRHI-GRM-P3: 91.02%), with higher ANIs with a strain found in the wheat stem sawfly *Cephus cinctus* (SiRHI-GRM-P1: 91.83%; SiRHI-GRM-P3: 91.92%) (Figure S1; Table S7). The close relationship between SiRHI-GRM-P1 and SiRHI-GRM-P3 is further supported by their high Amino acid identity (AAI) (99.73%). In contrast, their similarity to other members of the *S*. *ixodetis* clade is considerably lower, ranging from 61.08% to 91.91% (Figure 4B; Table S8). Overall, SiRHI-GRM-P1 and SiRHI-GRM-P3 possess 343 genes not found in other *S*. *ixodetis* strains or *Spiroplasma* species (of which 338 are shared by both SiRHI isolates, while the remaining five are specific to only one or the other) (Figure 4C). The majority of the genes specific to SiRHI MAGs encode hypothetical proteins whose function has not been determined (Table S9). Functional annotation of the two MAGs revealed the presence of various COG categories, with proportional distributions remaining broadly consistent between the two strains (Figure 4D; Table S10). Phylogenomic analysis based on 86 single-copy orthologs (SCOs; 26,303 unambiguously aligned amino acids) confirmed that SiRHI strains GRM-P1 and GRM-P3 cluster within the *S*. *ixodetis* clade, but further showed that their taxonomic position is sister relative to other members of the species *S*. *ixodetis* (Figure 4E). It suggests that SiRHI may either represent distinct strains within the *S*. *ixodetis* species or constitute a novel *Spiroplasma* species within the *S*. *ixodetis* clade. Pairwise AAI network analyses further supported this pattern, revealing that SiRHI and *S*. *ixodetis* endosymbionts form a clearly distinct clade, sharing less than 65% similarity with any other *Spiroplasma* species (Figure 5). In addition, ANI scores indicate that the *S*. *ixodetis* clade may comprise at least four species-level clusters (>95% ANI), highlighting the distinctiveness of SiRHI in comparison to *S*. *ixodetis* endosymbionts, *S*. *platyhelix*, and the *Spiroplasma* endosymbiont of the ant *Trachymyrmex septentrionalis* (Figure 5).

### Toxin and virulence factors in SiRHI metagenomes

**Figure 6:**
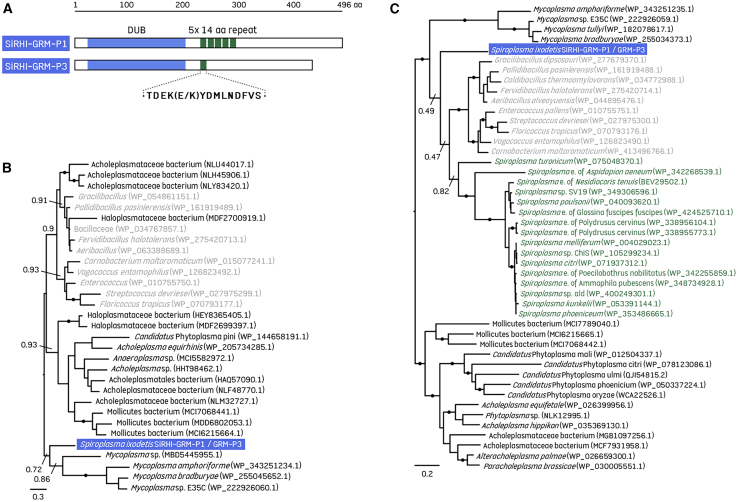
Putative virulence genes in SiRHI strains and other *Spiroplasma* strains and species (A) SiHRI-GRM-P1 and SiHRI-GRM-P3 MAGs encode an OTU-like cysteine protease domain-containing protein (a deubiquitinase [DUB]) likely to function in the intracellular host environment. The orthologs are identical except for a 14 amino acid (aa) domain that has expanded into five repeats in SiHRI-GRM-P1 and is present once in SiHRI-GRM-P3. SiRHI strains encode the molecular chaperone proteins GroES (B) and GroEL (C). Midpoint-rooted maximum likelihood phylograms constructed from amino acid sequences reveal sparcity of *GroE* genes in related taxa. *GroEL* and *GroES* are absent from other members of the *Spiroplasma ixodetis* clade but encoded by other *Spiroplasma* genomes (green tip labels) and two additional species. Filled circles on branches indicate support values > 0.90. *Spiroplasma* e., *Spiroplasma* endosymbiont. SiRHI-GRM-P1 and SiRHI-GRM-P3 MAGs are highlighted in blue. *Spiroplasma* sequences are shown in green text, while those from Mollicutes and non-Mollicutes are shown in black and gray text, respectively.

The SiRHI-GRM-P1 and SiRHI-GRM-P3 MAGs contain several candidate genes potentially involved in pathogenicity and host interaction (Figure 6; Table S11). Notably, we found six genes encoding ankyrin repeats (ANKs) and one encoding a deubiquitinase (DUB), which are key functional domains in the proteins that support the MK phenotype of *Spiroplasma*.^48^^,^^49^ Each of the ankyrin-repeat (ANK) proteins shows sequence similarity to ANK proteins of other arthropod-associated *Spiroplasma* species (Table S11). However, none are closely related to the ANK domains of the MK effector toxin, *Spaid*,^48^^,^^49^ nor are they paired with deubiquitinase (DUB) or other domains with predicted functions. The predicted DUB domain is an ovarian tumor (out)-like cysteine protease (Figure S2), a class also encoded by *Spaid* and shown to stabilize the MK toxin in eukaryotic cells.^49^ This protein does not have putative homologs in the genome of any arthropod-associated *Spiroplasma* species, but the DUB domain is distantly related to those encoded in some *Wolbachia* genomes (Figure S2). Aside from the DUB domain, the protein sequence shares no significant similarity to other proteins by blastp, though two *Spiroplasma* proteins and MSCRAMM family adhesins (microbial surface components recognizing adhesive matrix molecules, which mediate the initial attachment of bacteria to host tissues) of *Staphylococcus* species are among the very low similarity hits (Expect value > 1e^−5^). The protein is 496 and 440 amino acids in length in SiRHI-GRM-P1 and SiRHI-GRM-P3, respectively. The two orthologous genes are identical at the nucleotide level except for an indel of 168-bp in the center, located C-terminal of the DUB domain (Figure 6A). In SiRHI-GRM-P1 (locus 03720), this region comprises five copies of a 42-bp near perfect repeat, while in SiRHI-GRM-P3 (locus 00630) the 42-bp sequence element is present once. However, some other genes previously detected in *Spiroplasma* endosymbionts associated with arthropods, such as genes encoding ribosome-inactivating proteins (RIPs)^50^^,^^51^ and the CI factors *cifA*-*cifB*,^52^ were not detected in the SiRHI MAGs.

We identified eleven cell surface adhesin-like proteins in SiRHI-GRM-P1 and SiRHI-GRM-P3, all with homologs in other *S*. *ixodetis* clade genomes (Table S11), which can be implicated in virulence as observed in several *Spiroplasma* species associated with invertebrate disease.^53^^,^^54^ We also identified four putative surface lipoproteins (Table S11), which can have diverse functional roles including cell adhesion, invasion, and immune regulation.^55^ Homologs with moderate sequence divergence are present in other *S*. *ixodetis* clade genomes, and one lipoprotein, GRMP1_01065, also shares 55% aa identity with *Borrelia garinii* protein WP_210373045.

We detected single copy genes encoding the heat-shock proteins GroEL and GroES in SiRHI-GRM-P1 and SiRHI-GRM-P3 and performed phylogenetic analysis to compare these sequences (GroES, GRMP1_03335; GroEL, GRMP1_03330) with homologs in Mollicutes and relatives (Figures 6B and 6C). We found no homologs of *GroEL* in any genomes of the *S*. *ixodetis* clade sequenced to date, though there are orthologs in genomes of the *S*. *citri* clade, including *S*. *poulsonii* and pathogens of plants and invertebrates such as *S*. *citri* and *S*. *melliferum*. A *GroEL* homolog is also encoded by *S*. *turonicum*, a species affiliated with the *S*. *apis* clade and previously involved in a human infection case,^8^ as well as by an unclassified arthropod-associated strain (Figure 6C). Similarly, we found no homologs of *GroES* in any genomes of the *S*. *ixodetis* clade, though there are orthologs in few *Spiroplasma* genomes, mostly of the *S*. *citri* clade (Figure 6B). Phylogenetic trees of *GroEL* and *GroES* show similar topologies, placing SiRHI with moderate to low bootstrap support between *Mycoplasma* and non-Mollicutes outgroup taxa. Tracing the evolutionary origins of these genes in SiRHI remains difficult, largely due to the limited representation of closely related GroE operon genes. Nonetheless, the consistent absence of the GroE operon across all *S*. *ixodetis* clade genomes suggests that the SiRHI ancestor acquired the operon via horizontal gene transfer from an unknown bacterial donor. Notably, the GroE operon is identical between SiRHI-GRM-P1 and SiRHI-GRM-P3, consistent with a single horizontal acquisition event. However, only 800–1000 bp of identical flanking sequence is shared in either direction, suggesting that subsequent genomic rearrangements have occurred differentially across the SiRHI strains. Interestingly, a gene encoding the competence protein ComEC/Rec2, typically involved in DNA uptake, is located adjacent to the GroE operon, further supporting the hypothesis that this region was acquired via horizontal gene transfer.

## Discussion

This study identifies SiRHI as an emerging group of clinically relevant human pathogens, which form a distinct sublineage within the *S*. *ixodetis* clade. We identified 14, possibly 25, independent cases diagnosed between 1997 and 2024, all of which have thus far been reported exclusively in Europe. We also observed that SiRHI infections exhibit two distinct clinical syndromes based on patient age. In neonates and infants, infections cause severe ocular infections, leading to inflammatory cataracts that carry a high risk of amblyopia resulting in irreversible visual impairment.^1^^,^^2^^,^^3^^,^^4^ In adults, however, it manifests as acute febrile illness and non-specific systemic infections characterized by fever, thrombocytopenia, and increase in liver transaminases, without ocular disorder.^7^^,^^10^ These distinct clinical syndromes do not appear to be strain-specific, as the SiRHI-SWD strain was detected in both neonates or infants with ocular disorders and in adults with systemic infections, whereas the SiRHI-GRM strain was only detected in neonates and infants with ocular disorders.

We identified a wide diversity of *S*. *ixodetis* strains naturally circulating in 22 tick species, including species that commonly bite humans, domestic animals and wildlife, yet none of the characterized strains were genetically identical to the SiRHI strains. These findings suggest that *S*. *ixodetis* strains identified to date in ticks may not pose a risk to human health, as no vertebrate infections have ever been attributed to them, despite their frequent occurrence, often at high prevalence levels (10%–30%), in tick populations, particularly across Europe.^33^^,^^34^^,^^38^^,^^56^ Similarly, none of the *S*. *ixodetis* strains characterized so far from other arthropod hosts were found to be genetically identical to the SiRHI strains. This observation suggests either that SiRHI is not associated with arthropod transmission, or that the relevant vector species have not yet been investigated. If SiRHI strains are vector-borne, their apparent absence from the examined species suggests that they may either exist at lower prevalence than other *S*. *ixodetis* strains or circulate in alternative vector species or populations that have not yet been sampled. However, the identification of human infections in at least four, possibly five, European countries indicates that while these infections may be rare, they are geographically widespread. These observations indicate that the ecological contexts supporting the persistence and transmission of SiRHI strains are not unique to, nor confined within, a limited geographic region in Europe. Additionally, *S*. *ixodetis* naturally infects a broad spectrum of arthropods beyond ticks, including butterflies, ladybugs, and aphids,^24^^,^^28^^,^^38^^,^^42^^,^^43^^,^^44^ thereby raising the possibility that non-hematophagous arthropods may also serve as potential sources of human infection. Alternatively, human exposure might occur through direct contact with infected vertebrates, such as birds or small mammals that could serve as natural hosts, or indirectly with environments contaminated by infected animals. However, to date, there is no evidence of infection by SiRHI in other vertebrate hosts than humans. It is also likely that humans represent an evolutionary dead end for SiRHI, with infections occurring only as rare spillover events from its natural hosts. Indeed, SiRHI may be capable of persisting and disseminating within ecosystems without dependence on human hosts. The detection of the two SiRHI strains in neonates further raises the possibility of vertical transmission in humans, with mothers first becoming infected and subsequently transmitting the pathogen during pregnancy or birth.^1^^,^^2^^,^^3^ This also aligns with observations of SiRHI infections in neonates and infants with no history of tick exposure or outdoor activities.^1^^,^^2^^,^^3^

Phylogenetic analyses further highlight that the SiRHI strains fall within the *S*. *ixodetis* clade, sharing a common origin with arthropod-associated strains. Yet, SiRHI are less host-restricted and may cross species barriers to infect humans, resulting in the emergence of a new human infectious disease. Interestingly, the SiRHI genomes exhibit key genomic features shared with arthropod-associated *S*. *ixodetis* strains, confirming their common evolutionary origin. They encode protein domains found in characterized *Spiroplasma* toxins, such as the MK toxin *Spaid*, and commonly associated with endosymbiotic *Spiroplasma* species, including ANKs and DUBs. Although their specific roles in SiRHI remain unclear, ANKs, while also found in free-living bacteria, are notably enriched in the genomes of endosymbionts of arthropods,^57^^,^^58^^,^^59^ where they are thought to mediate diverse host interactions, including the modulation of cell death and immune responses.^60^ In *S*. *poulsonii*, ANK domains are involved in *Spaid*-mediated MK in *Drosophila* fruit flies^48^ and in other endosymbiont of arthropods, such *Wolbachia*, they have been found in numerous genes inducing, or highly suspected to induce, manipulative reproduction.^59^^,^^61^^,^^62^^,^^63^^,^^64^ Similarly, DUBs are key bacterial adaptation that promote their persistence within host cells: They are specialized proteases that regulate the ubiquitin system, a complex post-translational modification pathway found exclusively in eukaryotes, thereby disrupting protein degradation pathways.^65^ DUBs have been implicated in the mechanisms of CI and MK, suggesting a potential dual role in both intracellular survival and the manipulation of arthropod reproduction by endosymbionts such as *Spiroplasma*^48^^,^^64^^,^^66^ and *Wolbachia*.^63^^,^^67^^,^^68^

The chaperonins GroEL and GroES represent the most distinctive genomic features of SiRHI strains compared to arthropod-associated *S*. *ixodetis* and other *Spiroplasma* species. In bacteria with reduced genomes, GroEL is often highly expressed and plays a crucial role in mitigating the deleterious effects of elevated mutational load associated with genome reduction.^69^ In other Mollicutes, the patchy distribution of GroEL and GroES across virulent *Mycoplasma* species has also been hypothesized to reflect a role in promoting pathogenic phenotypes beyond their traditional housekeeping functions.^70^ Indeed, in various bacterial pathogens, GroEL has been directly implicated in host cell adhesion, immune modulation, and biofilm formation, which enhance host colonization and immune evasion.^71^ Moreover, the phylogenetic affinity of their GroEL and GroES sequences with homologs from Mollicutes and Firmicutes, and the presence of the competence gene *ComEC*/*Rec2* adjacent to the GroE operon together support the hypothesis that the SiRHI ancestor acquired this operon via horizontal gene transfer. Such a genetic acquisition may have been a critical evolutionary event, endowing SiRHI strains with both enhanced proteostasis under host-induced stress and additional virulence-associated capabilities that support human infection.

In conclusion, these findings suggest that the potential public health implications of SiRHI warrant further consideration. The distinct clinical symptoms and broad geographic occurrence suggest that SiRHI strains have undergone specific adaptations enabling cross-species transmission from currently unidentified arthropod hosts. Yet, the ecological reservoirs, transmission routes, and epidemiological dynamics of these pathogens remain poorly understood. Future research should prioritize the identification of vectors or reservoirs, clarify the possibility of vertical transmission in humans, and functionally characterize candidate virulence factors.

### Limitations of the study

To date, detection of SiRHI has been opportunistic, so prevalence, distribution, and the full spectrum of human infection remain uncertain. We have not yet identified any infected arthropods or animals, so the natural hosts of the SiRHI agents (and the routes by which humans become infected) remain unknown. Functional predictions of potential virulence factors are based on metagenomic investigations and await experimental validation. However, future investigations that bring together clinical, field, genomic, and experimental approaches have now the potential to clarify the ecology and zoonotic potential of SiRHI.

## Resource availability

### Lead contact

Requests for further information and resources should be directed to and will be fulfilled by the lead contact, Olivier Duron (olivier.duron@ird.fr).

### Materials availability

This study did not produce any new unique reagents.

### Data and code availability

• New gene sequences obtained in this study have been deposited in GenBank (NCBI: https://www.ncbi.nlm.nih.gov) with accession numbers PV583616-PV583682 (16S rRNA), PV644860-PV644927 (*rpoB*), PV644792-PV644859 (*dnaK*), PV644996-PV645059 (*gyrA*), and PV644928-PV644995 (*EpsG*). Additionally, the raw paired-end read datasets, along with single metagenome-assembled genomes (MAGs), are available as an archive SRA (Sequence Read Archive) under the Bioproject PRJNA1288376 and accession numbers JBQOLY000000000 (SiHRI-GRM-P1 MAG; TaxID: 3447963) and JBQOLZ000000000 (SiHRI-GRM-P3 MAG; TaxID: 3447964), all accessible in GenBank.
• All original code used for the genomic and phylogenetic analyses is available on GitHub (https://github.com/mariebuysse/Spiroplasma-human-infections_ms).
• Any additional information required to reanalyze the data reported in this paper are available from the lead contact upon request.

## Acknowledgments

We acknowledge the platform ISO 9001 certified IRD i-Trop HPC (South Green Platform; www.southgreen.fr) at IRD Montpellier for providing HPC resources that have contributed to the research results reported in this study. We are also grateful to Heylen Dieter, Maxime Duhayon, Nanet Fabri, Aitor Garcia Vozmediano, Mats van Gestel, Frédéric Jourdain, Sabrina Krief, Camille Lacroux, Karen McCoy, Olivier Plantard, Arnaud Robert, Frédéric Stachurski, Céline Teplitsky, Helene Verheyden, Laurence Vial, and Bronislava Vichova for facilitating access to tick samples. The *S*. *ixodetis* tick cell line samples were obtained from the Tick Cell Biobank at the University of Liverpool. This work was funded by French Agence Nationale de la Recherche (ANR, France, ref. ANR-21-CE02-0002 and ANR-25-CE02-7068, Laboratoire d’Excellence CEBA ANR-10-LABX-25-01 and 10.13039/100017605LabEx CeMEB ANR-10-LABX-04-01), the 10.13039/501100008222University of Montpellier (KIM RIVE [Key Initiative Montpellier: Risks and Vectors] and 10.13039/501100021565MUSE [Montpellier University of Excellence]), the Région Occitanie (Key challenge RIVOC), the United States 10.13039/100000001National Science Foundation (grant no. 2144270), the United Kingdom 10.13039/501100000268Biotechnology and Biological Sciences Research Council (grant no. BB/P024270/1), and the 10.13039/100010269Wellcome Trust (grant no. 223743/Z/21/Z).

## Author contributions

M. Buysse and O.D. designed the study. A.G., C.W., T.S., J.B., N.F., W.A.L., and A.S. characterized the clinical cases and collected human biosamples. M. Bruley, H.S., L.B.-S., M. Buysse, J.A., and J.G. identified infections in tick samples. M. Buysse, M.J.B., and J.A. conducted the metagenomic analyses. M. Buysse and O.D. wrote the draft of the manuscript. All authors contributed to and approved the final version of the manuscript.

## Declaration of interests

The authors declare no conflict of interest.

## Declaration of generative AI and AI-assisted technologies in the writing process

The authors acknowledge the use of ChatGPT for language editing support. After using this tool, the authors reviewed and edited the content as needed and take full responsibility for the content of the published article.

## STAR★Methods

### Key resources table

**Table:** 

REAGENT or RESOURCE	SOURCE	IDENTIFIER
**Critical commercial assays**		

DNeasy Blood and Tissue Kit	Quiagen	Cat#69504
Nextera XT DNA Library Preparation Kit	Illumina	Cat#FC-131-1024

**Deposited data**		

*Spiroplasma* multi-locus sequences	This study	NCBI accession numbers: PV583616-PV583682 (16S rRNA), PV644860-PV644927 (*rpoB*), PV644792-PV644859 (*dnaK*), PV644996-PV645059 (*gyrA*), and PV644928-PV644995 (*EpsG*); https://www.ncbi.nlm.nih.gov/
*Spiroplasma* metagenome-assembled genome GRM-P1	This study	NCBI accession number: JBQOLY000000000 (SiHRI-GRM-P1 MAG; TaxID: 3447963); Bioproject PRJNA1288376; https://www.ncbi.nlm.nih.gov/
*Spiroplasma* metagenome-assembled genome GRM-P3	This study	NCBI accession number: JBQOLZ000000000 (SiHRI-GRM-P3 MAG; TaxID: 3447964); Bioproject PRJNA1288376; https://www.ncbi.nlm.nih.gov/

**Software and algorithms**		

Chromas Lite	Technelysium	https://technelysium.com.au/wp/chromas/
MAFFT	Katoh et al.^72^	https://www.mafft.cbrc.jp/alignment/software
MEGA	Tamura et al.^73^	https://www.megasoftware.net
NCBI Genome Browser (GenBank)	_	https://www.ncbi.nlm.nih.gov/gdv/
FastQC	Andrews.^74^	https://www.bioinformatics.babraham.ac.uk/projects/fastqc/
cutadapt	Didion et al.^75^	https://cutadapt.readthedocs.io/en/
MEGAHIT	Li et al.^76^	https://github.com/voutcn/megahit
Concoct	Alneberg et al.^77^	https://github.com/BinPro/CONCOCT
anvi’o	Eren et al.^78^	https://anvio.org/
NCBI BLAST tool	_	https://blast.ncbi.nlm.nih.gov/Blast.cgi
QUAST	Gurevich et al.^79^	https://quast.sourceforge.net/
miComplete	Hogoson et al.^80^	https://github.com/EricHugo/miComplete
Bakta	Shwengers et al.^81^	https://github.com/oschwengers/bakta
CGView	Stothard et al.^82^	https://cgview.ca/
fastANI	Jain et al.^83^	https://github.com/ParBLiSS/FastANI
“heatmaply” R package	Galili et al.^84^	https://cran.r-project.org/web/packages/heatmaply/index.html
EzAAI	Kim et al.^85^	https://github.com/endixk/ezaai
Gephi	Bastian et al.^86^	https://gephi.org/
Pseudofinder	Syberg-Olsen et al.^87^	https://github.com/filip-husnik/pseudofinder
OrthoFinder	Emms et al.^88^	https://github.com/davidemms/OrthoFinder
‘ggVennDiagram’ R package	Gao et al.^89^	https://cran.r-project.org/web/packages/ggVennDiagram/readme/README.html
eggNOG-mapper	Cantalapiedra et al.^90^	http://eggnog-mapper.embl.de/
trimAl	Capella-Gutiérrez et al.^91^	https://trimal.readthedocs.io/en/latest/
AMAS	Borowiec et al.^92^	https://github.com/marekborowiec/AMAS
modeltest-ng	Darriba et al.^93^	https://github.com/ddarriba/modeltest
RAxML-NG	Kozlov et al.^94^	https://github.com/amkozlov/raxml-ng

**Other**		

Original code	This study	https://github.com/mariebuysse/Spiroplasma-human-infections_ms

### Experimental models and study participant details

#### Spiroplasma infections

DNA templates from six patients diagnosed with *Spiroplasma* infections were used for the present analysis (Tables 1 and S1). DNA templates were obtained from lens material collected during lensectomy procedures in a neonate and infants with cataracts and ocular inflammation in Germany (referred to as GRM-P1, GRM-P2, GRM-P3), and from blood samples of three adults in Sweden (SWD-P1, SWD-P2, SWD-P3) presenting with acute febrile illness similar to previous reported cases.^10^ Case reports detailing the clinical presentation, diagnostic process and DNA extraction have been previously published for three (GRM-P1, GRM-P2, GRM-P3) of the six patients, specifically one neonate and two infants.^3^^,^^4^ The diagnosis for the remaining four patients was established using the same protocols (Table S1). Our study followed the Strengthening the Reporting of Observational Studies in Epidemiology (STROBE) reporting guideline.

#### Ethics statement

Clinical samples from patients undergoing diagnostic evaluation for illness in Germany and Sweden were obtained during routine medical procedures. Most of these cases corresponded to previously documented case studies that had already been published. All patients provided written informed consent for the use of their pseudonymized clinical data in research, in accordance with applicable local ethical guidelines and regulations. The study analyzed only pre-existing, fully de-identified clinical specimens and associated pseudonymized data. No new samples were collected for the purposes of this study, and no direct patient interaction or access to identifiable personal information occurred. All procedures were conducted in compliance with relevant national regulations and institutional guidelines for research involving de-identified clinical datasets.

#### Tick samples

An additional set of 63 DNA templates was used for multi-locus typing analysis, consisting of field-collected nymphal and adult ticks (*n* = 55, representing six tick species), lab-reared ticks (*n* = 1, representing one tick species), and *S*. *ixodetis* isolated from naturally-infected ticks (*n* = 7, from four tick species) into tick cell lines (Table S3). Field tick samples included two major genera of hard ticks (family *Ixodidae*): *Ixodes* (five species) and *Dermacentor* (two species). Tick cell line samples comprised *S*. *ixodetis* isolated from naturally-infected embryonic or adult tissues of *I*. *ricinus*,^37^ *I*. *persulcatus*,^41^ *D*. *marginatus*, and *D*. *reticulatus*^40^ ticks and cultured continuously as intracellular infections in heterologous tick cell lines. Most tick samples originated from Europe, including specimens from multiple European countries (France, the Netherlands, Germany, Sweden, Belgium, Spain, Slovakia, and Russia), as well as some from outside Europe (Table S3). Each tick DNA template was obtained through extraction from whole tick bodies (individually or pooled) or pooled cultured tick cells, using the DNeasy Blood and Tissue Kit (QIAGEN, Hilden, Germany) according to the manufacturer’s instructions.

### Method details

#### Multi-locus typing of infections

Fragments of five genes (16S rRNA, *rpoB*, *dnaK*, *gyrA*, and *EpsG)* were amplified using semi-nested or nested PCR assays adapted from Binetruy et al.^38^ To obtain the full-length 16S rRNA gene of *Spiroplasma* from patient samples, specific primers and conditions were used as follows: a first PCR with the combination of 16S universal bacterial primers 16S_08F (5′-AGAGTTTGATYMTGGCTCAG-3′)^95^ and 16S_1507R (5′-TACCTTGTTACGACTTCA-3′)^96^ (annealing at 50°C, elongation of 2 min, 40 cycles), then a second PCR with the combination of primers designed for this study: Spixo_F0 (5′-AGTGGCGAACGGGTGAGTAACA-3′) and Spixo_R0 (5′-GCTACAGCACTGCCTCATGGCA-3′) (annealing at 52°C, elongation of 1 min and 30 s, 35 cycles). To prevent possible contamination, first and second PCR runs were physically separated from one another in entirely separate rooms. Negative (water) controls were included in each PCR assay. All PCR products were visualized through electrophoresis in a 1.5% agarose gel. All amplicons were purified and sequenced in both directions (EUROFINS, Luxembourg). Sequence chromatograms were cleaned with Chromas Lite (http://www.technelysium.com.au/chromas_lite.html), and alignments were performed using MAFFT (v7.525),^72^ then visualized with the MEGA software (v11.0.13).^73^

Strains of *S*. *ixodetis* were formally characterized based on single gene and concatenated gene sequence identity in 16S rRNA, *rpoB*, *dnaK*, *gyrA*, and *EpsG* nucleotide alignments. Sequence identity analyses included data from: (1) the *Spiroplasma* infections identified in the six human patients genetically characterized in this study, as well as 16S rRNA sequences available in GenBank for other *Spiroplasma* infection cases reported in humans (Table S1); (2) the *S*. *ixodetis* strains from field tick samples and tick cell lines characterized in this study (Table S3); (3) *S*. *ixodetis* strains from additional arthropod species available on GenBank (Table S4); and (4) the *Spiroplasma* genomes available on GenBank (Table S5).

#### Genome sequencing, assembly, and annotation

*Spiroplasma* MAGs were obtained by sequencing DNA extracts from infected patient samples (Table S1) using the Illumina HiSeq 2500 technology and after a library preparation performed with Nextera XT DNA Library Preparation Kit (Illumina, USA). The library preparation and the sequencing were performed by the platform MGX (Montpellier, France). The raw paired-end read quality was evaluated with FastQC (v0.11.9)^74^ and the reads were further trimmed via cutadapt (v3.1).^75^ We obtained 258,629,776 and 229,936,030 paired-end reads from lensectomy samples of an infant (GRM-P1) and a neonate (GRM-P3), but no high-quality paired-end reads from the samples of the four other patients (GRM-P2, SWD-P1, SWD-P2, and SWD-P3). MAGs from Illumina paired-end reads were assembled using MEGAHIT (v1.2.9) with default parameters.^76^ Contigs were further automatically clustered into genomes using the algorithm Concoct (v1.1.0)^77^ coupled with taxonomy tools implemented in the anvi’o pipeline.^78^ Assignation of genomes was next confirmed using the online NCBI BLAST tool (https://blast.ncbi.nlm.nih.gov/Blast.cgi). The quality assessment and the completeness of each genome were estimated using QUAST (v4.6.3)^79^ and miComplete (-hmms Bact105) (v1.1.1).^80^ The newly obtained genomes were annotated using Bakta (v1.9.3)^81^ using translation table 4. Graphical representations of newly sequenced genomes were produced using CGView (v1.5).^82^

#### Genomic content, metabolic pathway analysis, and virulence factor identification

To characterize newly obtained *Spiroplasma* MAGs associated with human infection cases, a nucleic sequence identity matrix was calculated between the two MAGs (GRM-P1 and GRM-P3) and multiple *Spiroplasma* isolate genomes from the *S*. *ixodetis* clade using fastANI (v1.34),^83^ then visualized using the “heatmaply” R package.^84^ In addition, an amino acid identity matrix was calculated between the two MAGs and the genomes of representative *Spiroplasma* isolates and species from the *S*. *ixodetis*, *S*. *apis*, *S*. *mirum*, and *S*. *citri* clades using EzAAI (v1.2.2–0),^85^ then visualized in the same way. Networks of ANI and AAI results were produced in Gephi (v.0.10.1)^86^ using Frutcherman Reingold layout and with edges filtered for >0.65 AAI and >0.95 ANI scores. Pseudogene prediction was performed using Pseudofinder (v1.0)^87^ on each MAG. The genomic datasets were filtered to exclude pseudogenes from the subsequent analyses. To compare the genomic content of obtained MAGs and representative *Spiroplasma* genomes, Single Copy Orthologs (SCOs) were identified using OrthoFinder (v2.5.4),^88^ then SCO lists were retrieved on a R environment to build a Venn diagram using the ‘ggVennDiagram’ R package.^89^ The functional annotation of proteins into Clusters of Orthologous Genes (COGs) was predicted using eggNOG-mapper (v2.1.12),^90^^,^^97^ then the COGs’ presence was plotted with an in-house R script. Putative virulence factor searches were done using tblastn with an Expect value cutoff of 1e^−5^. Query sequences including ANKs, DUBs, RIPs and *cifA-cifB* were collected from annotated *Spiroplasma* and other bacterial genomes. HMMER and hhpred were used for functional domain predictions.

#### Phylogenetics

Phylogenetic reconstructions were performed using either the 16S rRNA dataset, the MLST dataset, or the genomic dataset. For the 16S rRNA and MLST dataset, nucleotide sequences were aligned with MAFFT and filtered to remove poorly-aligned positions using trimAl (v1.5.rev0).^91^ For the MLST dataset, the *rpoB*, *dnaK*, and *gyrA* sequences were concatenated using AMAS (v1.01).^92^ For the genomic dataset, SCOs shared between the obtained MAGs and representative *Spiroplasma* genomes were identified using OrthoFinder, then processed with MAFFT, trimAl, and AMAS. The most suitable evolutionary model for each dataset was determined with modeltest-ng (v0.1.7)^93^ according to the corrected Akaike Information Criterion (AICc). Maximum likelihood (ML) trees were then constructed using RAxML-NG (v1.0.0)^94^ with 1,000 bootstrap replicates. *GroEL* and *GroES* amino acid sequences were aligned with MAFFT (v7.490) and the phylogram was constructed with FastTree (v2.1.11) using the JTT model.

### Quantification and statistical analysis

No quantification or statistical analyses were performed in this study.

## Supplemental information

Document S1. Figures S1 and S2 and Tables S6 and S11

Table S1. List of reported human cases of infection with *Spiroplasma*, including details on major associated symptoms, *Spiroplasma* detection method, treatment and report of tick bites Isolates SiRHI-GRM-P1 to GRM-P3 and SiRHI-SWD-P1 to SWD-P3 are from the six patients analyzed in this study. 16S rRNA∗: the positions of the sequence in relation to the *S*. *ixodetis* strain Y32 reference sequence, along with the accession number. CRP: C-reactive. ALT: alanine. AST: aspartate

Table S2. 16S rRNA nucleotide pairwise identity matrix between the SiHRI cases and other *Spiroplasma* species

Table S3. Detailed list and origin of *Spiroplasma*-infected tick specimens examined in this study, including accession numbers of obtained 16S rRNA, *rpoB*, *dnaK*, *gyrA*, and *EpsG* gene sequences

Table S4. List of tick and other arthropod species with Spiroplasma ixodetis 16S rRNA, *rpoB*, *dnaK*, *gyrA*, and *EpsG* sequences available on public databases

Table S5. List and main genomic features of *Spiroplasma* genomes available on public databases

Table S7. Acid nucleic identity matrix between the SiHRI MAGs and other *Spiroplasma* species from the Ixodetis clade

Table S8. Amino acid identity matrix between the SiHRI MAGs and other Spiroplasma species

Table S9. List of genes specific to SiHRI MAGs

Table S10. COG categories description of SiRHI MAGs
