# Supplementary material for: A human-associated Spiroplasma ixodetis lineage responsible for infantile cataracts and adult febrile illness
Source: iScience. 2026 Mar 6;29(4):115233. doi: 10.1016/j.isci.2026.115233 (PMC13049210; doi:10.1016/j.isci.2026.115233)
Supplement: Document S1. Figures S1 and S2 and Tables S6 and S11 [file mmc1.pdf]

iScience, Volume *29*

Supplemental information

A human-associated *Spiroplasma ixodetis* lineage responsible for infantile cataracts and adult febrile illness

Marie Buysse, Matthew J. Ballinger, Marjorie Bruley, Julien Amoros, Justine Grillet, Navid Farassat, Annerose Serr, Wolf Alexander Lagrèze, Christine Wennerås, Anna Grankvist, Thomas Schön, Jonas Berglund, Lesley Bell-Sakyi, Hein Sprong, and Olivier Duron

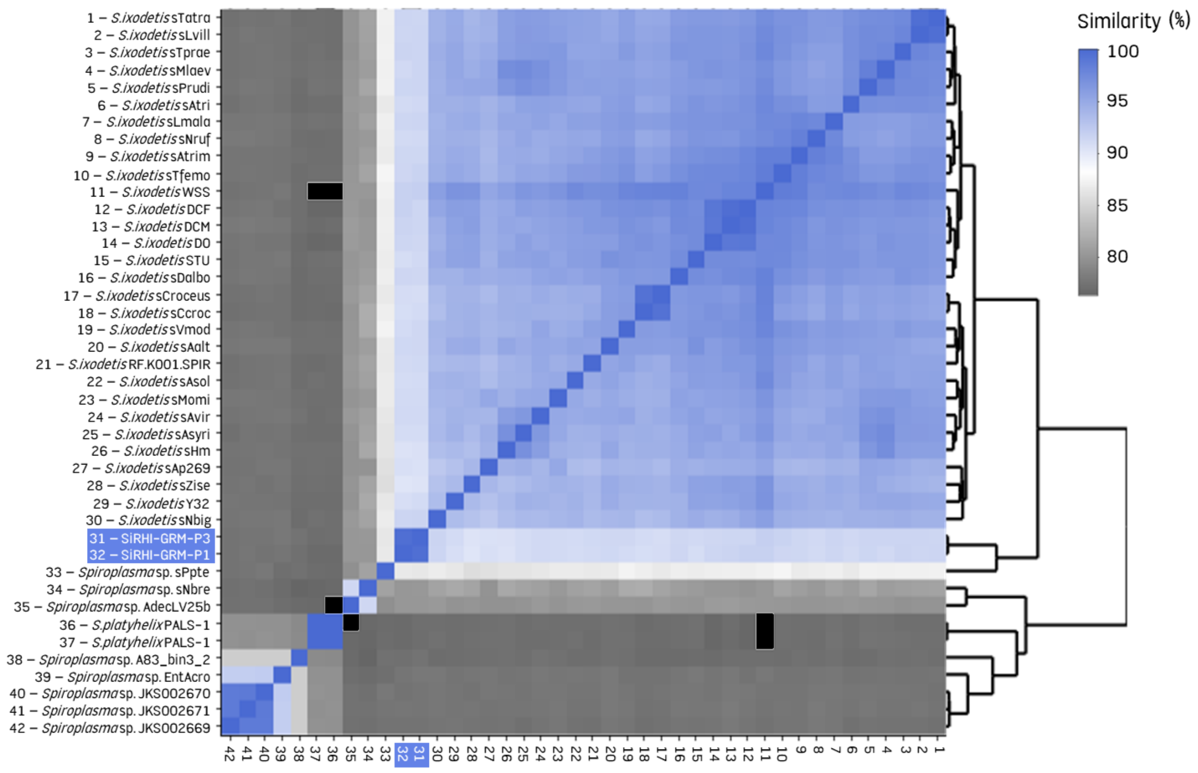

Figure S1. ANI matrix with values represented by color, accompanied by a cladogram based on these values. SiRHI-GRM-P1 and SiRHI-GRM-P3 MAGs are highlighted in blue. The black boxes correspond to values below the calculation threshold of the fastANI tool.

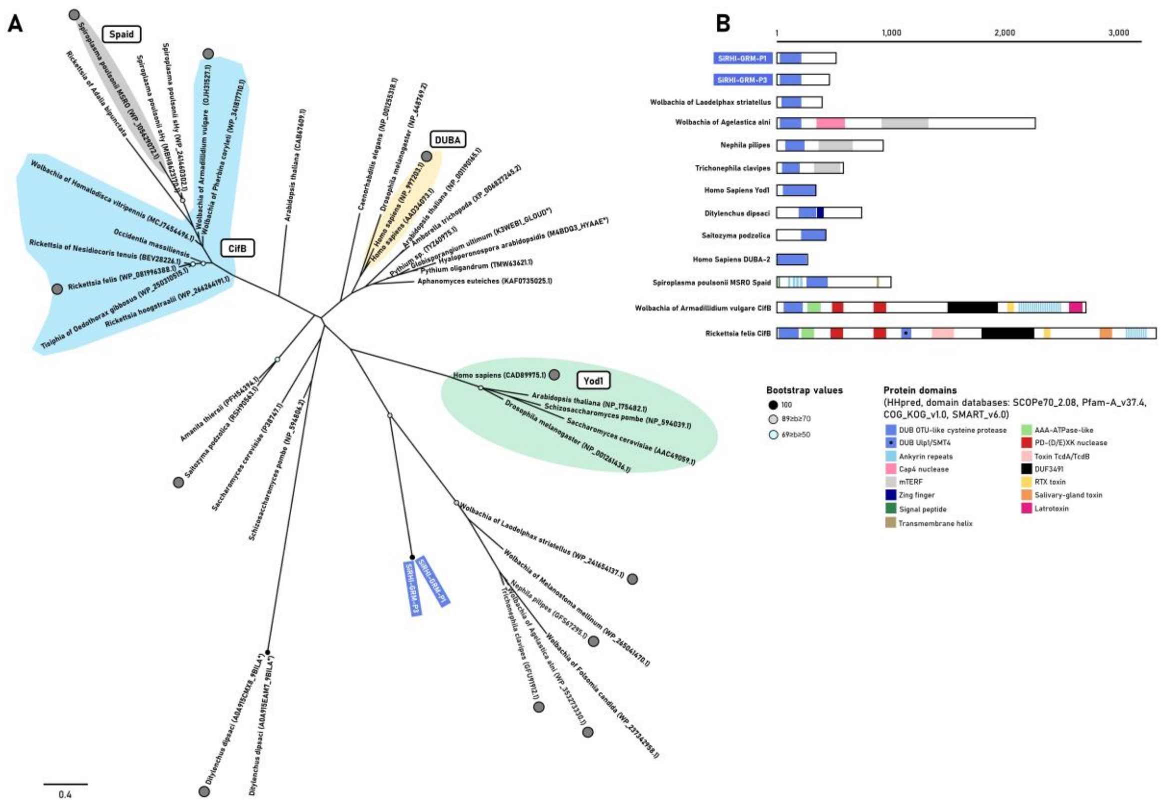

Figure S2. Proteins containing OTU-like cysteine protease domains. (**A**) Maximum likelihood phylogeny inferred from amino acid sequences of the OTU-like cysteine protease (best-fit evolutionary model: LG+G4). The tree is midpoint-rooted. Numbers on each node represent bootstrap support percentages from 1,000 replicates, with only values greater than 50% shown. The scale bar corresponds to the mean number of substitutions per site. SiRHI-GRM-P1 and SiRHI-GRM-P3 proteins are highlighted in blue. The known protein groups Spaid, CifB, DUBA and Yod1 are colored. (**B**) Comparative protein domain architecture and amino acid lenghts of the sequences marked with a grey circle in panel (A).

Table S6. Main genomic features of SiHRI MAGs.

	Genome size (bp)	Contigs number	N50	L50	Completeness (%)	Redundacy	GC rate (%)	CDSs	Pseudogenes	Coding density (%)	Coverage values
SiRHI-GRM-P1	1,246,387	176	11.503	34	78.10	1.1585	22.81	1317	108	64.8	517.19X
SiRHI-GRM-P3	1,233,617	202	9.944	41	77.14	1.1481	22.84	1312	107	64.4	1892.68X

Table S11. List of toxin and virulence genes in SiHRI MAGs and representative *Spiroplasma* genomes.

Putative lipoproteins

GRM-P1 contig	GRM-P1 locus	length (aa)	hhpred hit	hhpred description	hhpred prob	hhpred expect	Signal peptide score	Lipoprotein signal score	P3 homolog locus	P3 homolog status
108	_03925	240	8EM4_A	Low-density lipoprotein receptor-related protein 2	99.19	2.40E-08	0.0004	0.9996	_00820	identical
32	_01065	131	8H1R_C	Lipoprotein signal tag	96	0.021	0.0181	0.9819	contig_66; no locus tag	pseudogene
156	_05575	410	4A2L_B	Two-component system sensor histidine kinase/response	99.96	3.60E-26	0.01968	0.8017	_01120	identical
25	_00910	332	4A2L_B	Two-component system sensor histidine kinase/response	99.91	5.80E-21	0.125	0.8747	_04890	identical

Adhesin-like proteins

GRM-P1 contig	GRM-P1 locus	length (aa)	Signal peptide score	Lipoprotein signal score	P3 homolog locus	P3 homolog status
98	_03555	876	0.6683	0.0095	_03760	incomplete
58	_02110	135	0	0	_02315	identical
77	_02845	181	0	0	_01155	complete; 96.5% nt identity
80	_02920	308	0	0	_02140	partial; identical
82	_02940	136	0	0	_00950	extended; identical
85	_03125	259	0	0	_01225	extended; 97.1% nt identity
102	_03747	138	0	0	_05410	extended; 96.4% nt identity
131	_05000	306	0	0	_01245	incomplete; 99.5% nt identity
133	_05025	180	0	0	_00300	identical
160	_05695	160	0	0	_01155	extended; 99.4% nt identity
192	_06810	395	0	0	no locus tag	partial; 100% nt identity

Ankyrin domain proteins						
**GRM-P1 contig**	**GRM-P1 locus**	**length (aa)**	**Signal peptide score**	**Lipoprotein signal score**	**P3 homolog locus**	**P3 homolog status**
4	_00300	113	0	0	_03075	identical
72	_02675	429	0	0	no locus tag; contig_86	partial; identical; contig end
117	_04455	65	0	0	_03565	identical
155	_05565	394	0	0	_05170	identical
34	_01095	292	0	0	_03135	identical
153	_05415	151	0	0	_04255	identical
**OTU cysteine protease**						
**GRM-P1 contig**	**GRM-P1 locus**	**length (aa)**	**Signal peptide score**	**Lipoprotein signal score**	**P3 homolog locus**	**P3 homolog status**
101	_03720	496	0	0	_00630	168 bp indel
